# Persistent reservations against the premedical and medical curriculum

**DOI:** 10.1007/s40037-013-0047-2

**Published:** 2013-03-15

**Authors:** Vinay Prasad

**Affiliations:** Medical Oncology Branch, National Cancer Institute, National Institutes of Health, 10 Center Dr. 10/12N226, Bethesda, MD 20892 USA

Reflecting on medical education from the vantage of caring for patients, I am tempted to conclude that nearly every step is irrelevant for the next one. This is not a novel or profound observation. Emanuel [1] has argued that premedical curriculum (organic chemistry, calculus and physics) is not useful for clinical practice (nor most biomedical research). Previously, I have argued [2] that the first 2 years of medical school (biochemistry, molecular biology, neuroscience, physiology) are unnecessary for the second 2 years, and encourage a dangerous cognitive bias: subordinating evidence-based medicine to pathophysiological reasoning. This bias has been shown to play a key role in popularizing unproven medical practices [3], many of which are later overturned, a phenomenon called medical reversal [3]. Other critics have concluded rather bluntly that basic science education in the first 2 years of medical school is ‘a waste of time’ [4].

Similar criticism has been levelled against the Medical College Admissions Test, the United States Medical Licensing Exams, and field-specific medical board examinations. These tests have been criticized as: only weakly predicting medical school performance [5, 6], not being predictive of who does well in residency [7] (despite being used for that purpose), and not reflective of real world practice [8], respectively. Here, I wish to focus not on how we ended up in this situation—where every portion of professional training has been challenged (largely by following historical precedent)—nor on how training should be restructured (there have been countless suggestions)—but on the persistent arguments in favour of the status quo. Why do many believe we should not change our system of medical education, or worse, that we have already made the necessary changes?

## But, I learned critical thinking in organic chemistry

The quintessential rebuttal to arguments that some portion of medical training is antiquated, esoteric and no longer relevant for practising clinicians is this one: but, organic chemistry teaches critical thinking. In fact, we can make this a generalizable statement: But, <insert your favourite class here> was where I learned <critical thinking, logical reasoning, thinking on the fly, multitasking, visualizing problems, the ability to memorize large volumes of information, recall under pressure, etc.>. A version of this argument is ubiquitous. In response to Emanuel’s critique of the premedical curriculum [1], one physician writes, ‘I would not so hastily dismiss organic chemistry…. In a well-taught course, the specific facts… are secondary to the skills of pattern recognition, systematic analysis of complex problems, thinking in three dimensions…’ Another group, defending the basic sciences, writes, ‘Study in the sciences contributes to training in logical thinking and developing an inquisitive mindset that is integral to effective clinical reasoning [9].’

Although I can see its appeal, this argument is misguided. It is not fair to concede that the content of a course is irrelevant, but argue that, in the process, students learn some alternative, valuable skill. Because, if it is the case that hard work, critical thinking or <insert your favourite skill here> is desirable among physicians, then we should teach that skill explicitly, or study the alternative ways to acquire the skill, and pick the path that best achieves that goal or does so in the least time. For instance, if the ‘systemic analysis of complex problems’ is a tool that good doctors need (and I tend to agree), is it organic chemistry, or a course in logic, or political science, or ancient Greek philosophy, or a novel course studying real-life medical problems that best inculcates this skill? And furthermore, how many years does it take? These are testable and important questions.

## But, without a mechanistic understanding, doctors will just be technicians

Proponents of the basic sciences argue that the knowledge imparted in such courses is precisely what elevates physicians above the role of mere technicians, and to the status of professionals. Professionals understand the mechanistic rationale of why a therapy or diagnostic test makes sense, while technicians merely know under what circumstances they should be used. Technicians are perfectly able to function in the day-to-day management of even complicated issues, but when there are situations that do not neatly fit a predefined mould, only a professional can devise a plan, so the argument goes. Indeed, making decisions in the face of uncertainly may very well be the mark of a professional; however, a mechanistic understanding of science is just one way someone might navigate this.

In some cases, mechanistic reasoning may be completely mistaken. For instance, for a patient with heart failure caused by ischaemia, a mechanistic understanding of science would lead one to conclude that statin therapy should be beneficial. However, when directly examined this was not the case [10]. In our review of contradicted medical practices, we found that the most common reason practices were adopted and later contradicted was precisely because they were supported only by a mechanistic belief they would work [2]. Uncertainty is one of the core dilemmas faced by physicians, and we must have ways to reason in these situations. However, whether basic science is the best guiding salvo is far from certain. Elsewhere I have detailed other examples where mechanistic reasoning has mislead [2], and provided alternate ways to adjudicate uncertainty [3].

## But, we don’t know who the next J. Michael Bishop will be

Another common argument in favour of the status quo (that I frequently hear from the science faculty) is that we do not know who the next J. Michael Bishop, Roderick McKinnon, Harold Varmus, or <insert your favourite MD Nobel laureate here> will be. Thus, we must continue to teach molecular biology, biochemistry, and related subjects so that, if a student in our ranks is destined to become a research giant, he or she will not have been deprived of the opportunity for early exposure to fundamental biomedical research.

It is easy to see why this argument lacks muster. Must every medical student be exposed to years of basic science coursework so that a select few may choose to focus on it? Surely, we can imagine less time-consuming screening tests. In a similar fashion, we also do not know who the next William Carlos Williams, Rivka Galchen, Khaled Hosseini, Michael Crichton or <insert your favourite doctor novelist here> will be, but we do not make all physicians practice short story writing (although some might argue that it would foster empathy [11]).

The simple solution is to allow time for students to pursue diverse interests, without burdening those disinterested, while having a core medical curriculum address the common thread among MD graduates—providing care for patients. Most medical schools have the opportunity for electives in the final year; those who are interested can take basic science courses.

## But, basic science is the foundation for medical education

Lastly, we come to the most common defence of basic science in premedical and medical education: that it is the foundation, period. Basic science remains as important as it did in Flexner’s time. One group reaches this conclusion after internal discussion, ‘Our findings revealed a consensus with Flexner’s view in 1910—regarding both the basic sciences (anatomy, biochemistry, neuroscience, physiology, microbiology, immunology, pathology, and pharmacology) that a preclinical medical education should include and the fact that these sciences remain vital foundations of medical practice [9].’ Another writer concurs, ‘Knowledge of the applications of biochemistry, molecular biology, and genetics in the practice of medicine has been and continues to be a vital part of medical students’ and continuing education [12].

The assertion that basic science is the foundation of clinical medicine is common, and frequently made by those who teach the courses in question. However, claiming that some coursework is the foundation for future practice does not make sense, if, in that future practice, the prior coursework is never used [13]. Simply because it was mandated does not make biochemistry a foundation.

## But, we’ve already made changes

The final objection is that we have already made dramatic changes to medical education. A 1983 report [14] called for a movement away from detail-oriented medical education, a 1992 report asked that basic science be made relevant by being incorporated throughout all 4 years [15]. Many medical schools have made changes to the curriculum in response to these calls; however, such reforms often reorganize basic science curricula (into organ systems, for instance), and fail to question their fundamental value. A high profile article on medical education reform suggests, rather meekly, that other competencies be added to the ‘traditional biomedical content’ [16]. ‘For this reason, others have called such reforms, ‘cosmetic’ in nature [4].

The case against organic chemistry, physics, calculus (premed), biochemistry, molecular biology, (prolonged, i.e. 20 weeks of) anatomy, neuroscience, embryology, and even histology has been made repeatedly in the medical education literature. Despite the many arguments from practising physicians that this subject matter is not used daily in clinical medicine, and, despite the further criticism that such bottom up learning sanctions cognitive biases [2], there has been little real change in medical education, as these courses continue to be mandated. Several groups [1, 4, 15] note that other competencies—statistics, bioethics, communication, and health policy—are more deserving of a place in the medical curriculum than most basic science. But this argument is overly generous, and likely intended as a compromise. All classes should be evaluated by the same standard of relevance, and reasons for omitting basic science go beyond considerations of what might fill its place. Here, I have tried to articulate the common objections in favour of the status quo. None carry strong appeal. Regardless of what we add to medical education, we must cut subjects that are not used by the vast majority of graduating physicians.
